# Supplementary material for: Altered microRNA Transcriptome in Cultured Human Liver Cells upon Infection with Ebola Virus
Source: Int J Mol Sci. 2021 Apr 6;22(7):3792. doi: 10.3390/ijms22073792 (PMC8038836; doi:10.3390/ijms22073792)
Supplement: Supplementary file 1 [file ijms-22-03792-s001.zip › Supplementary_File/C_ GO_Analysis_Results/16-30nt_go_Mayinga-24h-Huh7_vs_Control-24h-Huh7_down.mature_mirna_targets/MF_Pvalue_tree.pdf]

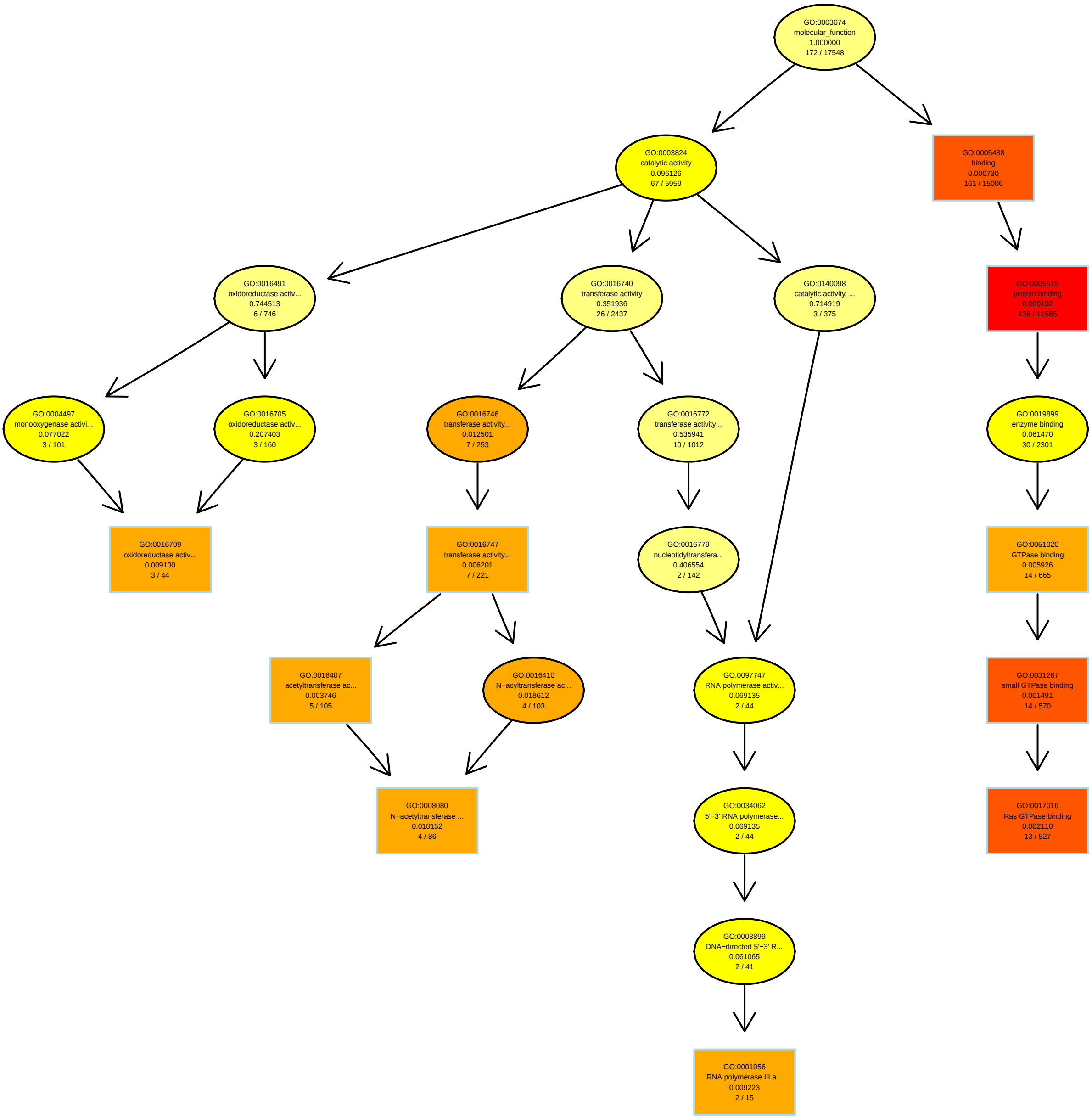
GO:0003674 molecular_function 1.000000 172 / 17548
GO:0003824 catalytic activity 0.096126 67 / 5959
GO:0005488 binding 0.000730 161 / 15006
GO:0016491 oxidoreductase activ... 0.744513 6 / 746
GO:0016740 transferase activity 0.351936 26 / 2437
GO:0140098 catalytic activity, ... 0.714919 3 / 375
GO:0005515 protein binding 0.000102 136 / 11565
GO:0004497 monooxygenase activi... 0.077022 3 / 101
GO:0016705 oxidoreductase activ... 0.207403 3 / 160
GO:0016746 transferase activity... 0.012501 7 / 253
GO:0016772 transferase activity... 0.535941 10 / 1012
GO:0019899 enzyme binding 0.061470 30 / 2301
GO:0016709 oxidoreductase activ... 0.009130 3 / 44
GO:0016747 transferase activity... 0.006201 7 / 221
GO:0016779 nucleotidyltransfera... 0.406554 2 / 142
GO:0051020 GTPase binding 0.005926 14 / 665
GO:0016407 acetyltransferase ac... 0.003746 5 / 105
GO:0016410 N−acyltransferase ac... 0.018612 4 / 103
GO:0097747 RNA polymerase activ... 0.069135 2 / 44
GO:0031267 small GTPase binding 0.001491 14 / 570
GO:0008080 N−acetyltransferase ... 0.010152 4 / 86
GO:0034062 5'−3' RNA polymerase... 0.069135 2 / 44
GO:0017016 Ras GTPase binding 0.002110 13 / 527
GO:0003899 DNA−directed 5'−3' R... 0.061065 2 / 41
GO:0001056 RNA polymerase III a... 0.009223 2 / 15
